# Hydrogen sulfide is a novel regulator implicated in glucocorticoids-inhibited bone formation

**DOI:** 10.18632/aging.102269

**Published:** 2019-09-16

**Authors:** Jun Ma, Changgui Shi, Zhongyang Liu, Bin Han, Lei Guo, Lei Zhu, Tianwen Ye

**Affiliations:** 1 Department of Orthopedic Surgery, Changzheng Hospital, Second Military Medical University, Shanghai, China; 2 Department of Orthopedic Surgery, The 72nd Military Hospital of PLA, Huzhou, China; 3 Department of Orthopedic Surgery, Chinese PLA General Hospital, Beijing, China; 4 Shanghai Key Laboratory for Bone and Joint Diseases, Shanghai Institute of Orthopaedics and Traumatology, Shanghai Ruijin Hospital, Shanghai Jiaotong University School of Medicine, Shanghai, China

**Keywords:** hydrogen sulfide, glucocorticoids, osteoporosis, osteoblast

## Abstract

Glucocorticoids contribute to the increased incidence of secondary osteoporosis. Hydrogen sulfide (H_2_S) is a gasotransmitter and plays an essential role in bone metabolism. In this study, we investigated the therapeutic effects of H_2_S on glucocorticoid-induced osteoporosis (GIO). We found that dexamethasone (Dex) decreased serum H_2_S and two key H_2_S-generating enzymes in the bone marrow *in vivo*, cystathione b-synthase and cystathione g-lyase. Treatment of H_2_S-donor GYY4137 in rat significantly relieved the inhibitory effect of Dex on bone formation. Dex inhibited osteoblasts proliferation and osteogenic differentiation and decreased the expressions of the two H_2_S-generating enzymes. Further investigation showed that H_2_S was involved in Dex-mediated osteoblasts proliferation, differentiation, and apoptosis. Mechanistically, GYY4137 promoted osteoblastogenesis by activating Wnt signaling through increased production of the Wnt ligands. In comparison, the blockage of Wnt/β-catenin signaling pathway significantly alleviated the effect of H_2_S on osteoblasts. In conclusion, the restoration of H_2_S levels is a potential novel therapeutic approach for GIO.

## INTRODUCTION

Glucocorticoids (GCs) are widely used for the treatment of inflammatory and immune disorders, such as rheumatoid arthritis, systemic lupus erythematosus, and asthma [1, 2]. However, prolonged GCs administration could result in bone loss and increased bone fragility, osteoporosis, and fracture [3, 4]. Indeed, Glucocorticoid-induced osteoporosis (GIO) is currently the main leading cause of secondary osteoporosis, and nearly 30% of the patients receiving long-term GCs suffered from bone fractures [5]. Osteoblasts, the main target cells in the pathological process of osteoporosis, play an important role in bone formation and growth [6, 7]. Previous evidence suggests that GCs could directly disrupt osteoblasts differentiation and promote osteoblasts apoptosis [8–12].

Hydrogen sulfide (H_2_S), a colorless and poisonous gas with a foul odor of rotten eggs, has been recognized as an important gasotransmitter regulating various physiological or pathological cellular functions [13–15]. Endogenous H_2_S is generated from cysteine, which is catalyzed by cystathionine b-synthase (CBS) and cystathionine c-lyase (CSE) [16]. H_2_S has been identified in several tissues, including the vasculature, kidney, heart, brain, nervous system, and lung [17]. H_2_S is also involved in the acquisition and preservation of bone mass [18–20]. H_2_S controls stem cells function by regulating Ca^2+^influx through Ca^2+^ channels, and deficiency of H_2_S impairs the osteogenic differentiation of the stem cells [18]. Recent studies revealed that H_2_S protects osteoblastic cells from H_2_O_2_ or Dex-induced cell injury [21, 22]. However, the molecular mechanism remains elusive and needs further investigation.

The Wnt/β-catenin signaling pathway plays a vital role in regulating osteoblasts differentiation and bone formation [23, 24]. In the absence of Wnt ligands, β-catenin forms a multiprotein complex with casein kinase 1(CK1), glycogen synthase kinase 3β (GSK3β), adenomatous polyposis coli (APC), and Axin. This process drives β-catenin ubiquitination and degradation [25, 26]. Whether Wnt/β-catenin signaling is involved in H_2_S-induced osteogenic differentiation is unknown.

Based on the capacity of H_2_S to regulate osteoblastogenesis, we hypothesized that GCs may impair the endogenous synthesis of H_2_S and exogenous H_2_S may play a role in GCs-induced bone loss. In the present study, we found that pharmacological restoration of normal levels of H_2_S prevented Dex-induced osteoporosis by enhancing bone formation. In addition, H_2_S protected osteoblasts from Dex through activating Wnt signaling. Our results demonstrated the therapeutic potential of H_2_S for the treatment of GCs-induced osteoporosis.

## RESULTS

### Production of H_2_S was decreased in Dex-induced osteoporosis

To investigate whether Dex affects bone formation and systemic H_2_S levels, 3-month-old rats were injected with Dex for 4 weeks. Rats were euthanized and serum H_2_S levels were assessed. BMSCs were harvested and cultured for 1 week to assess the levels of CBS and CSE mRNAs. Femurs cancellous bone was analyzed by μCT.

**Figure 1:**
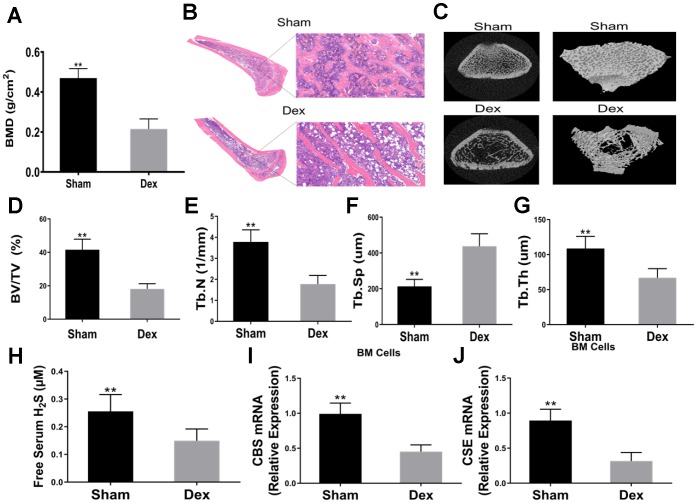
**Dexamethasone induced osteoporosis and downregulated serum H2S and mRNA expressions of CBS and CSE in BM SCs.** (**A**) BMD in the distal end of the intact femurs of each experimental group. n=5, **p<0.01. (**B**) Hematoxylin-eosin staining was performed to identify histological structures of the distal end of the intact femurs of rats injected with Dex for 4 weeks. Scale bars are 50 μm. (**C**) Representative figures of micro-CT analysis of the distal end of the intact femurs of rats treated with Dex. (**D**) BV/TV in the distal end of the intact femurs of each experimental group. n=5, **p<0.01. (**E**) Tb.N in the distal end of the intact femurs of each experimental group. n=5, **p<0.01. (**F**) Tb.Th in the distal end of the intact femurs of each experimental group. n=5, **p<0.01. (**G**) Tb.Sp in the distal end of the intact femurs of each experimental group. n=5, **p<0.01. (**H**) Effects of Dex on serum H2S. n=5, **p<0.01. (**I**–**J**) Effects of Dex on mRNA expressions of CBS and CSE in BMSCs. n=5, **p<0.01.

The results revealed that the BMD of Dex-induced rats was significantly lower than that of the control group (Figure 1A). HE staining showed that Dex induced less amount of trabecular bones (Figure 1B). Representative μCT images of femur trabecular bone are shown in Figure 1C. Dex-treated rats had lower BV/TV than those of the sham group (Figure 1D). Analysis of the indices of the trabecular structure revealed that Tb.N, Tb.Th, and Tb.Sp were also differential in Dex-treated and vehicle-treated rats (Figure 1E–1G). Compared with the vehicle-treated rats, Dex-treated rats had lower levels of serum free H_2_S (Figure 1H). The mRNA expression of CBS and CSE in BMSCs were significantly lower in the Dex group than in the control group (Figure 1I–1J).

### Exogenous hydrogen sulfide alleviated osteoporosis induced by Dex

**Figure 2:**
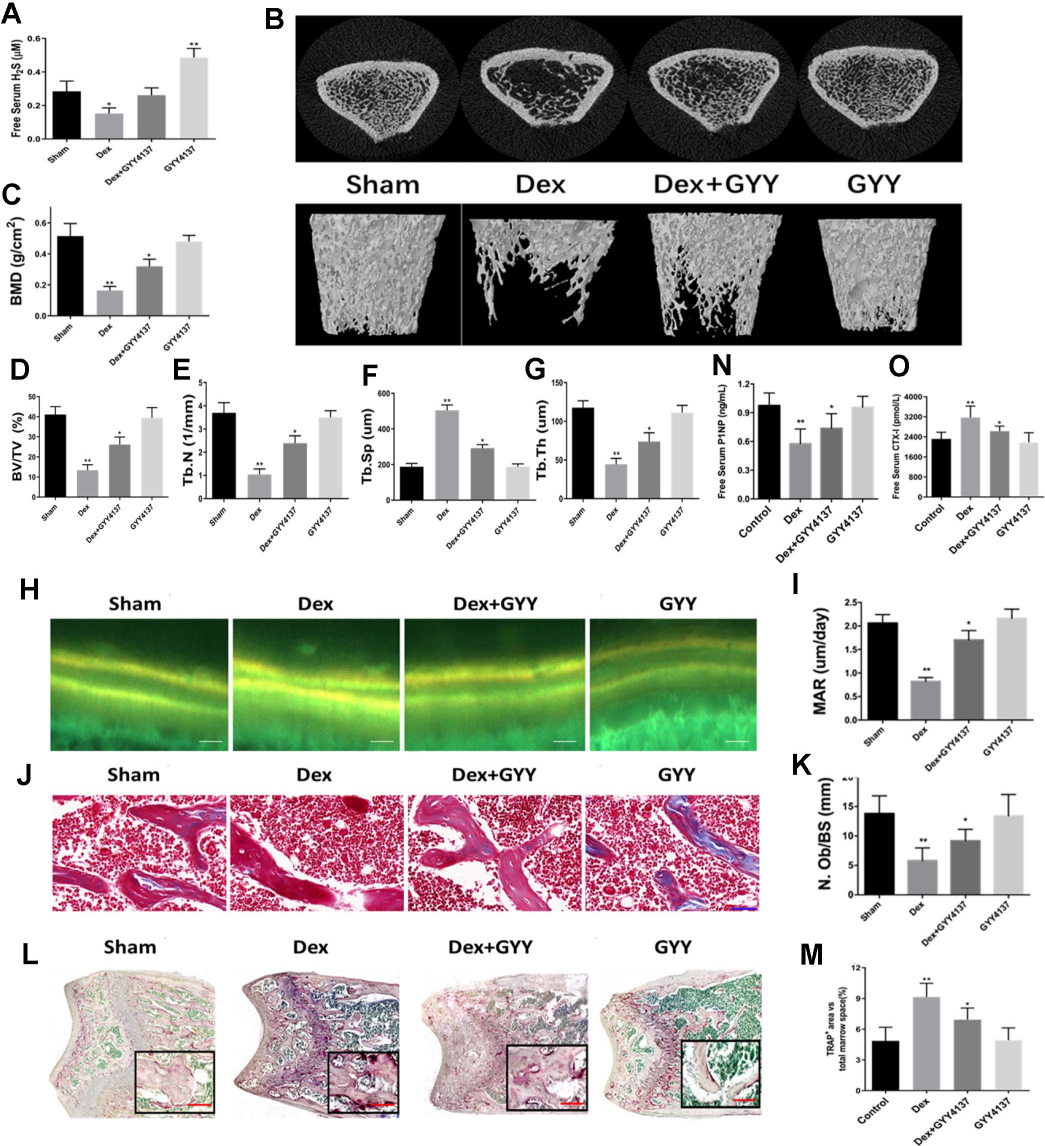
**Exogenous hydrogen sulfide alleviated osteoporosis induced by Dex.** (**A**) Serum levels of H2S, 8 weeks after treatment with GYY4137 per group. (**B**) 3D-image reconstruction of one representative femur per group. (**C**) BMD in the distal end of the intact femurs of each experimental group. (**C**) BMD, (**D**) BV/TV, (**E**) Tb.N, (**F**) Tb.Sp, and (**G**) Tb.Th in the distal end of the intact femurs of each experimental group. (**H**) Tetracycline labels observed by fluorescence light microscopy in the slices of the tibia of each experimental group. Scale bars are 50 μm. (**I**) Quantitative analysis of mineral apposition rate. Bone sections were fixed, decalcified, dehydrated, and sectioned. Masson trichromic staining (**J**) and TRAP staining (**L**) was shown. Scale bars are 50 μm for Masson and 200 μm for TRAP staining. Quantitative analysis of osteoblast number/bone surface (N.Ob/BS) (**K**) and osteoclast area/total marrow space (**M**). (**N** and **O**) Serum levels of CTX-I and P1NP after 8 weeks of treatment with GYY4137 in each group. n=5, *p<0.05, **p<0.01.

To investigate the role of H_2_S on GIO, the rats were divided into four groups. Rats in the control group (saline only) and Dex group (Dex intervention) were treated with either GYY4137 or vehicle. After 8 weeks of treatment, Dex significantly decreased serum free H_2_S levels. However, administration of GYY4137 attenuated this inhibitory effect (Figure 2A). The results demonstrated that GYY4137 was able to prevent sulfur deficiency caused by Dex.

Representative μCT images of femur trabecular bone from all four groups are displayed in Figure 2B. The results showed that Dex significantly decreased BMD, BV/TV, Tb.N, and Tb.Th, while increased Tb.Sp (Figure 2C–2G). Additionally, bone loss induced by Dex was attenuated by GYY4137 treatment (Figure 2C–2G).

To explore whether H_2_S could attenuate the Dex-inhibiting effects on bone formation, double-calcein labeling was assessed by repeated injection of tetracycline. The results showed that the MAR was significantly lower in Dex-treated rats than in the controls, which was attenuated by the addition of GYY4137 (Figure 2H–2I). Similar results were observed for the number of osteoblasts (N.Ob/BS), which was shown by Masson trichromic staining (Figure 2J–2K). Next, we analyzed the TRAP staining in the metaphyseal area of femur bone sections (Figure 2L). The number of TRAP-positive cells was increased in Dex-treated rats compared with the control group. Administration of GYY4137 attenuated this increase (Figure 2M). In addition, the serum level of CTX-1 was significantly higher in the Dex-treated group compared to the sham group (Figure 2N), while the opposite finding was observed for the level of P1NP (Figure 2O). However, addition of GYY4137 significantly blocked the effects of Dex on CTX-1 and P1NP levels (Figure 2N–2O). Taken together, these results strongly suggested that GYY4137 could protect against the loss of bone mass induced by Dex.

### Dex decreased osteoblasts proliferation and osteogenic differentiation, blunted CSE and CBS expression

**Figure 3:**
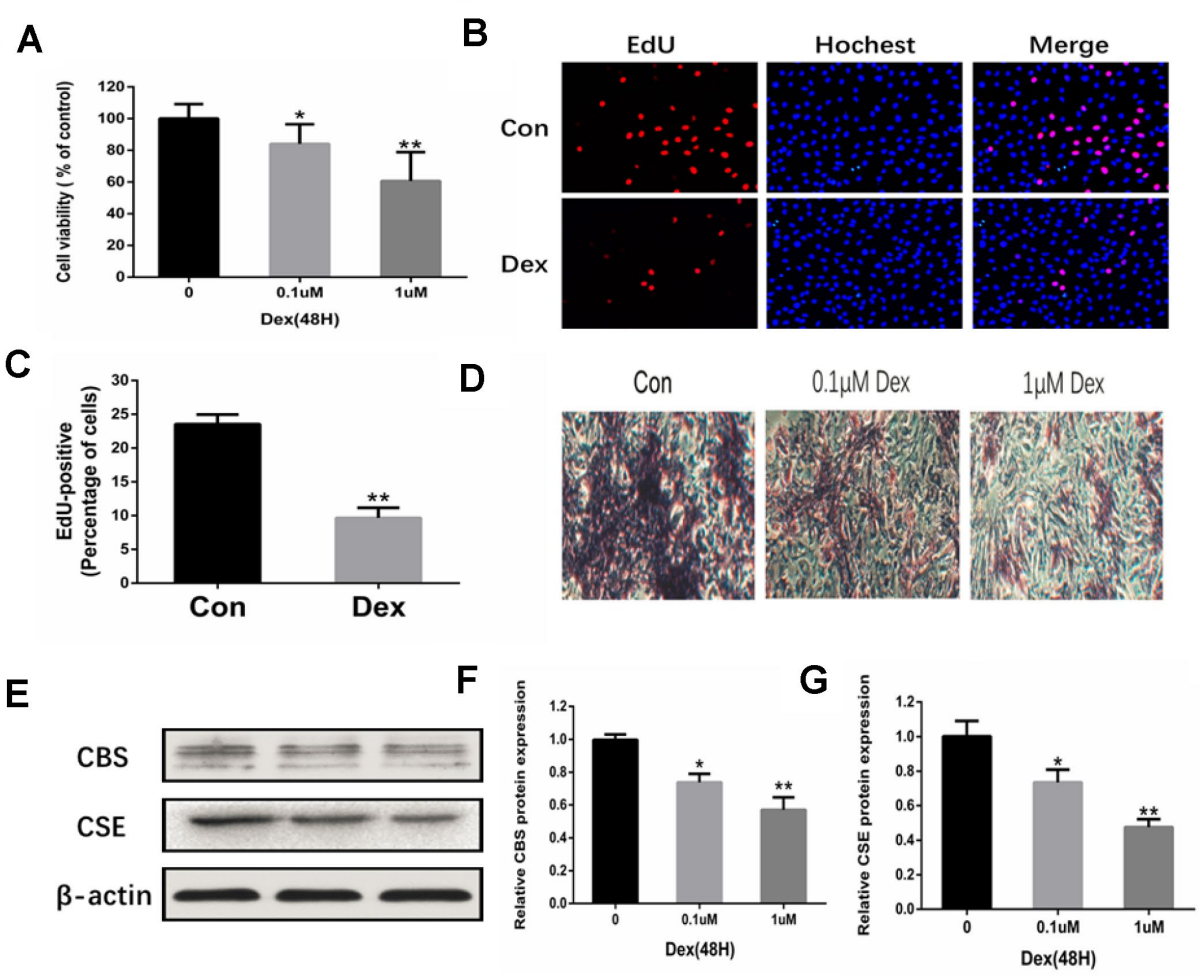
**Inhibition of osteoblast proliferation and expression of CBS and CSE by Dex.** (**A**) Cell viability of rat primary osteoblasts was measured by MTT after cells were treated with 10−7 and 10−6 M Dex for 48 h. n=3, **p<0.01. (**B**) Representative photomicrographs (x200) of EdU staining and corresponding total cell photomicrographs. Blue: Hoechst labeling of cell nuclei; red: EdU labeling of nuclei of proliferative cells. (**C**) Quantitative data showing the percentage of EdU-positive cells in different treatment groups (number of red vs number of blue nuclei). n=3, *p<0.05, **p<0.01. (**D**) Dex inhibited osteogenic differentiation of primary osteoblasts in a dose-dependent manner, as evidenced by changes in mineralized matrix formation (ARS staining, day 14) (x100). (**E**–**G**) Western blot analysis showing that Dex decreased the levels of CBS and CSE in primary osteoblasts in a dose-dependent manner. n=3, *p<0.05, **p<0.01.

To assess the effects of Dex on the proliferation and differentiation of osteoblasts, rat primary osteoblasts were incubated with different concentrations of Dex (0, 10^-7^ and 10^-6^ M) for 24 h. MTT assay was used to monitor cell viability. The results indicated that Dex decreased the viability of osteoblasts in a dose-dependent manner (Figure 3A). These results were further confirmed by EdU staining. Fewer osteoblasts with EdU-positive nuclei were observed in the Dex-treated group as compared to the control group (Figure 3B–3C). In addition, Dex inhibited the cell osteogenic potential, as evidenced by the ARS staining (14 days) (Figure 3D). Western blot analysis showed reduced expressions of CBS and CSE in the osteoblasts incubated with Dex in a dose-dependent manner (Figure 3E–3G).

### Hydrogen sulfide alleviated the inhibitory effects of Dex on osteoblast differentiation, proliferation, and survival

**Figure 4:**
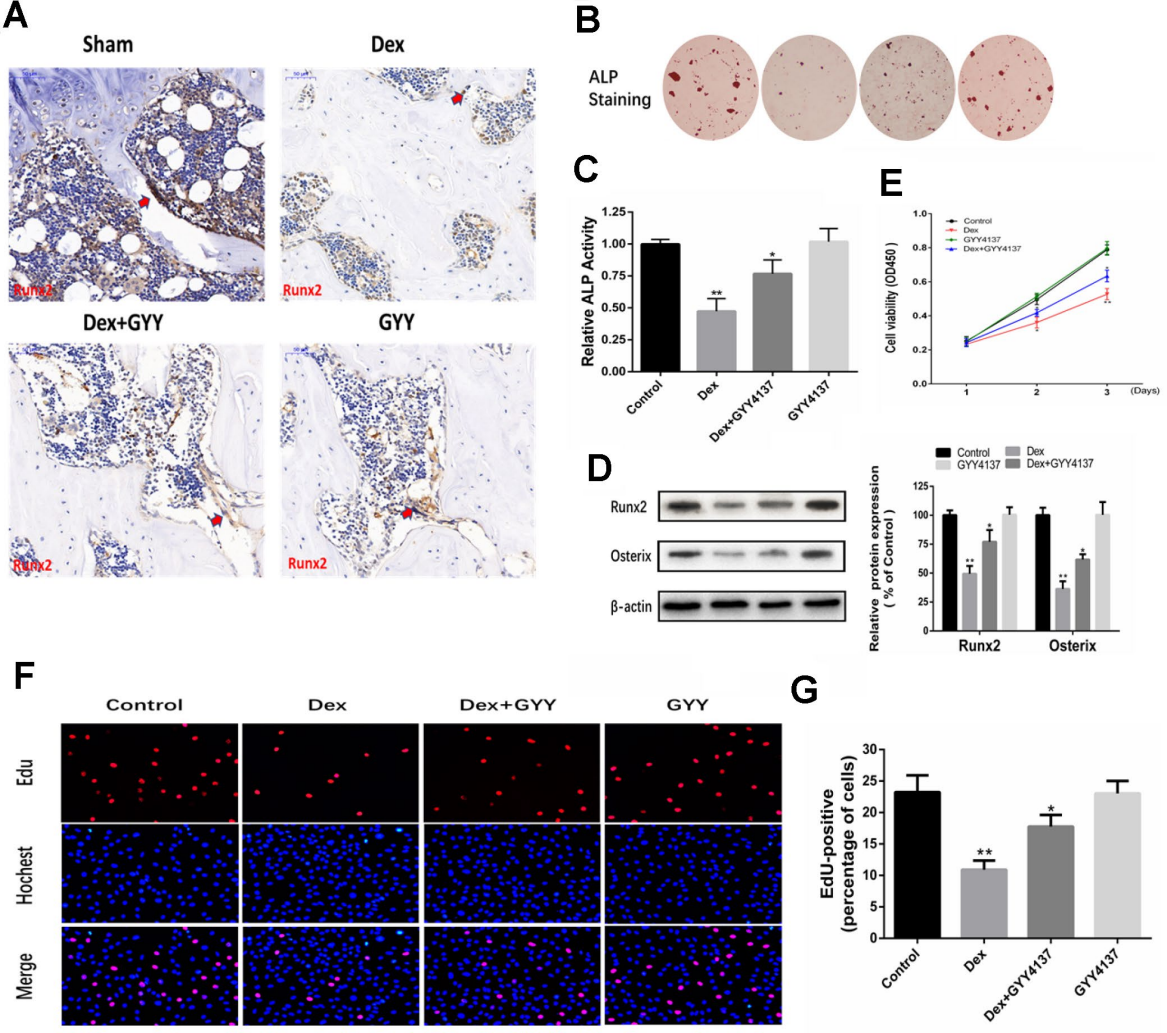
**Hydrogen sulfide alleviated the inhibitory effect of osteoblast differentiation, proliferation, and survival by Dex.** (**A**) Runx2 expression and localization (red arrow) in the distal end of intact femurs of each experimental group through immunohistochemistry. (Scale bars 50 μm). (**B**–**C**) As shown by ALP staining (day 7) (x20) and ALP activity detection (day 7), GYY4137 attenuated the effect of Dex on the inhibition of osteogenic differentiation in primary osteoblasts. (**D**) Western blot analysis of Runx2 and Osterix expression in rat primary osteoblasts pretreated with Dex and/or GYY4137. n=3, *p<0.05, **p<0.01. (**E**) The proliferation of rat primary osteoblasts cells was measured by CCK8 assay after cells were treated with Dex and/or GYY4137 from day 1 to day 3. n=3, *p<0.05, **p<0.01. (**F**) Representative photomicrographs (x200) of EdU staining (top panels) and corresponding total cell photomicrographs (middle panels). Blue: Hochest labeling of cell nuclei; red: EdU labeling of nuclei of proliferative cells. (**G**) Quantitative data showing the percentages of EdU-positive cells in different treatment groups (number of red versus number of blue nuclei). n=3, *p<0.05, **p<0.01.

The localization and expression of Runx2 were measured to examine the effect of GYY4137 on osteoblast differentiation. The results showed that Dex remarkably decreased the expression of Runx2. However, administration of GYY4137 attenuated this effect (Figure 4A). Furthermore, Dex decreased osteogenic differentiation, which was attenuated by GYY4137, as evidenced by ALP staining (Figure 4B). ALP activity was also decreased by Dex in a dose-dependent manner, which was attenuated by GYY4137 (Figure 4C). Western blot analysis showed that the master osteogenic transcription factors, Runx2 and Osterix, in the osteoblasts were significantly downregulated by Dex. In contrast, the protein levels were reversed in the presence of GYY4137 (Figure 4D). To delineate the role of GYY4137 in osteoblast proliferation, we assessed cell proliferation with CCK-8 assays. We found that GYY4137 at 100 μM produced significant protective effects against Dex-induced cell injury (Figure 4E). EdU assay by fluorescence microscopy further demonstrated that GYY4137 alleviated the inhibitory effects of Dex on osteoblast proliferation (Figure 4F–4G).

### Hydrogen sulfide protected osteoblasts against Dex-induced apoptosis

**Figure 5:**
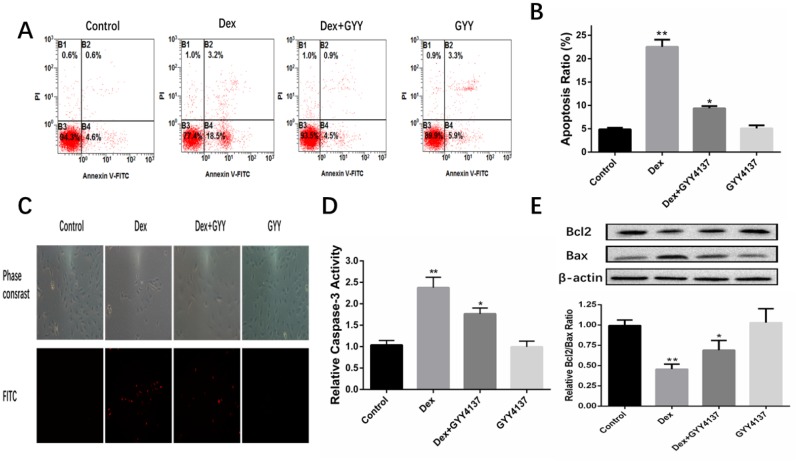
**GYY4137 protected osteoblasts against Dex-induced cell apoptosis.** (**A**) Cell apoptosis was detected by annexin V-fluorescein isothiocyanate/propidium iodide double staining and examined with a fluorescence-activated cell sorting flow cytometry analyzer. (**B**) The results of flow cytometric analysis are shown as percentages of positive. n=6, *p<0.05, **p<0.01. (**C**) Cell apoptosis was detected by Hoechst 33342 staining in cells treated with vehicle, Dex, Dex +GYY4137 and GYY4137. Cells with condensed or fragmented nuclei were identified as apoptosis cells and counted based on nuclear condensation or fragmentation. (**D**) Caspase-3 activity was measured in primary osteoblast pretreated with Dex or/and GYY4137.n=6, *p<0.05, **p<0.01. (**E**) BCL2 and BAX protein was analyzed by western blot for primary osteoblast pretreated with Dex or/and GYY4137. n=6, *p<0.05, **p<0.01.

Apoptotic progression was monitored in the osteoblasts via Hoechst 33342 staining assays and AnnexinV-FITC/PI double staining. The results indicated that Dex treatment induced condensed and fragmented nuclei, which is a characteristic of apoptosis. However, these changes were significantly relieved when the cells were pretreated with GYY4137 (Figure 5A–5C). Caspase-3 plays an important role in the process of apoptosis, and thus caspase-3 activity assay was used to measure apoptosis. A significant increase in caspase-3 activity was noticed in cells treated with Dex compared with the control group. GYY4137 significantly attenuated the increased caspase-3 activity (Figure 5D). Next, the levels of BCL2 and BAX were analyzed by Western blot. The results showed that Dex caused a reduction in the BCL2/BAX ratio in osteoblasts, but this decrease was relieved in cells pretreated with GYY4137 (Figure 5E). Taken together, these results implied that H_2_S may protect osteoblasts against Dex-induced cell apoptosis.

### Wnt/β-catenin pathway was involved in the protective effect of hydrogen sulfide against Dex-induced osteoblast activities

**Figure 6:**
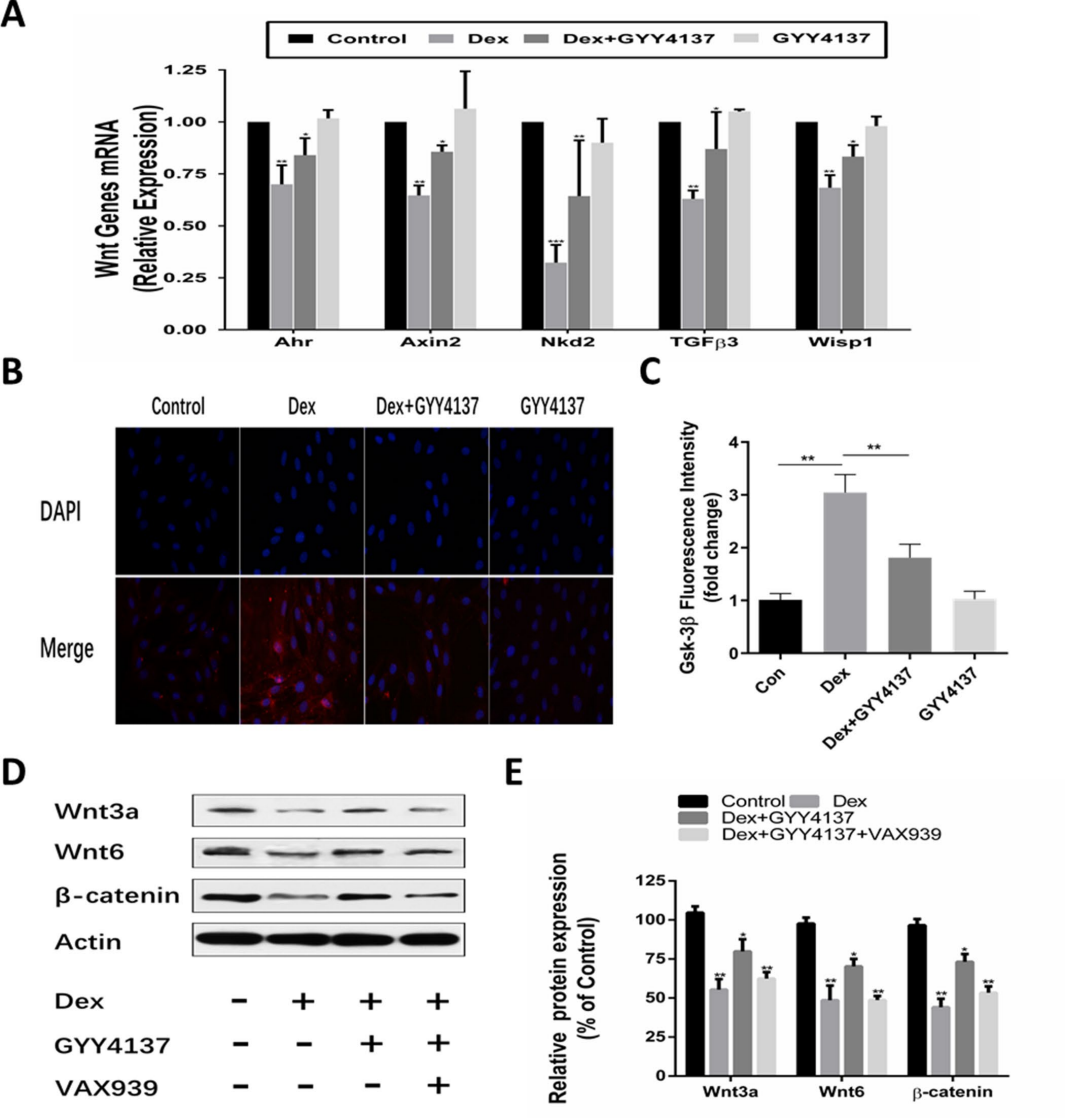
**Involvement of the Wnt/β-catenin pathway in the protective effect of GYY4137 against Dex-induced osteoblast activities.** (**A**) mRNA expression of Wnt-signaling target genes was measured in primary osteoblast pretreated with Dex or/and GYY4137. n=3, *p<0.05, **p<0.01. (**B**–**C**) Immunofluorescence staining of the osteoblast after a 2-day incubation with Dex or/and GYY4137. (**D**–**E**) Effect of the wnt/β-catenin inhibitor on the protein expression of wnt3a, wnt6, β-catenin at 48hr with GYY4137 pretreatment in Dex-induced osteoblast activities. n=3, *p<0.05, **p<0.01.

Osteoblast differentiation is regulated by Wnt activation. Our previous study found that Dex inhibited osteoblasts differentiation and proliferation by negatively regulating Wnt signaling. Thus, we investigated whether Wnt/β-catenin signaling pathway was involved in the effect of GYY4137 on GCs-mediated osteoblast function. The cells were pretreated with 100M GYY4137 or without GYY4137 for 30 min and then were treated with 1mol/L Dex or without Dex for 2 days. QRT-PCR analyses showed that the mRNA levels of sensitive markers of Wnt activation (i.e., Ahr, axin2, Nkd2, GFb3, and Wisp1) were all decreased in the osteoblasts exposed to Dex. However, pretreatment with GYY4137 markedly alleviated this inhibitory effect (Figure 6A). The immunofluorescence imaging revealed that treatment with Dex significantly elevated the fluorescence intensity of GSK-3β. Whereas pretreatment with GYY4137 significantly decreased the fluorescence intensity of GSK-3β (Figure 6B–6C). Next, we analyzed the expression of Wnt signaling specific proteins. The results showed that treatment with Dex significantly downregulated the protein expression of Wnt3a, Wnt6, and β-catenin. Pretreatment with GYY4137 significantly attenuated the inhibitory effect of Dex on the Wnt signaling pathway, and the effect of H_2_S was attenuated after pretreatment with a Wnt/β-catenin inhibitor (XAV939) (Figure 6D–6E).

### Knockdown of Wnt3a or Wnt6 abolished the effect of GYY4137 on Dex-treated osteoblast proliferation and differentiation

**Figure 7:**
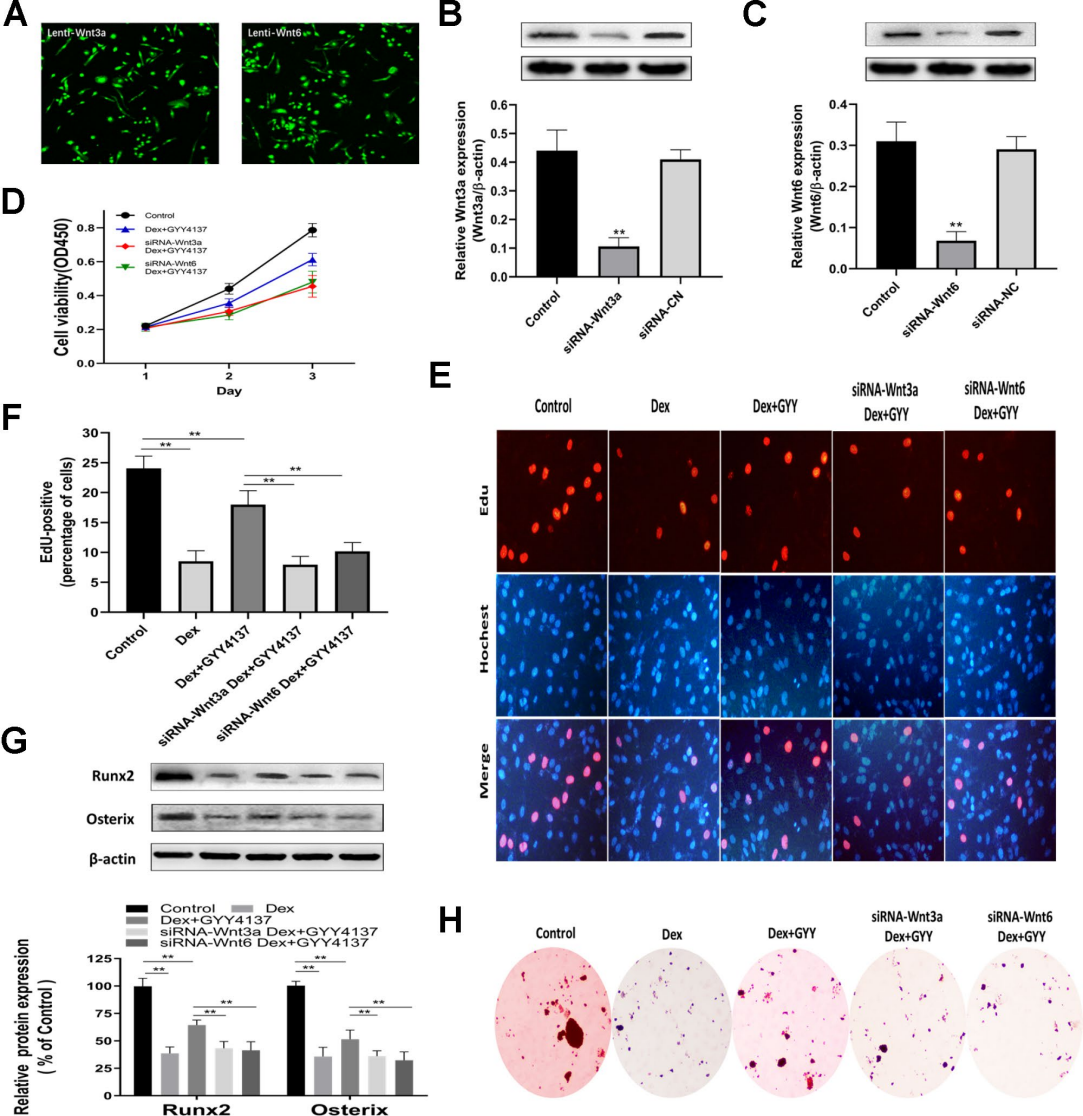
**Knockdown of Wnt3a or Wnt6 abolished the GYY4137 effect on Dex-treated osteoblast proliferation and differentiation.** (**A**) All cells expressed GFP, showing that the cells were infected by lentivirus. (**B**–**C**) Verified Wnt3a or Wnt6 knockdown effect by lentivirus-mediated transduction of primary culture osteoclasts precursors. n=3, **p<0.01. (**D**) The proliferation of rat primary osteoblasts cells was measured by CCK8 assay after the cells were infected by lentivirus. (**E**) Representative photomicrographs (x200) of EdU staining and corresponding total cell photomicrographs. Blue: Hoechst labeling of cell nuclei; red: EdU labeling of nuclei of proliferative cells. (**F**) Quantitative data showing the percentages of EdU-positive cells in different treatment groups (number of red versus number of blue nuclei). n=3, **p<0.01. (**G**) Western blot analysis of Runx2 and Osterix expression in rat primary osteoblasts of each group. n=3, **p<0.01. (**H**) ALP staining was measured in primary osteoblast per group.

To explore the role of Wnt/β-catenin pathway in the effect of GYY4137 on Dex-treated osteoblasts, the levels of Wnt3a and Wnt6 were separately silenced with lentiviral constructs encoding shRNA targeting Wnt3a (Lenti-shRNA-Wnt3a) and lentiviral constructs encoding shRNA targeting Wnt6 (Lenti-shRNA-Wnt6). All the cells expressed GFP, indicating that the cells were infected by lentivirus and also expressed shRNA (Figure 7A). Western blot analysis of Wnt3a and Wnt6 expression confirmed that the deletion of Wnt3a or Wnt6 was effective (Figure 7B–7C). CCK-8 assays revealed that the knockdown of Wnt3a or Wnt6 abolished the effect of GYY4137 on Dex-treated osteoblast proliferation (Figure 7D). These results were further confirmed by EdU staining (Figure 7E–7F). In addition, the expressions of osteogenic marker genes, including Runx2 and Osterix, were significantly affected in osteoblast cells transfected with Lenti-shRNA-Wnt3a or Lenti-shRNA-Wnt6 for 48 h (Figure 7G). The results were further supported by ALP staining (Figure 7H). Taken together, we found that knockdown of Wnt3a or Wnt6 abolished the effect of GYY4137 on Dex-treated osteoblast proliferation and differentiation.

## DISCUSSION

Long-term GCs therapy could cause severe osteoporosis [5]. Several studies have indicated that GCs inhibit osteoblast proliferation and promote osteoblast apoptosis [27, 28]. However, the molecular mechanisms remain elusive. As a novel signaling molecule and an important cryoprotectant, H_2_S protects MC3T3-E1 cells against GCS-induced oxidative damage [15, 22, 29] This study further confirmed the protective effects of H2S against GC-induced damages in the osteoblasts *in vivo* and *in vitro*.

In the *in vivo* part, our data showed that intraperitoneal injections of Dex blunted the production of H_2_S in BMSCs by downregulating the key enzymes CBS and CSE. Our findings were consistent with earlier reports, which demonstrated that decreased production of H_2_S might intensify the effects of oxidative stress in bone [21, 22]. GYY4137 is a slow-releasing H_2_S donor that has been widely used to investigate the role of H_2_S in biological processes [30–32]. We used GYY4137 treatment to prevent Dex-induced bone loss. GYY4137 recovered the levels of free H_2_S and prevented femoral trabecular and cortical bone loss. The presence of a relationship between H_2_S level and bone volume suggests that H_2_S is a potential candidate for bone metabolism, and exogenous supplement of H_2_S could prevent Dex-induced bone loss by enhancing bone formation and inhibiting bone absorption.

In the *in vitro* part, we examined the effects of Dex on osteoblastic proliferation and differentiation. In line with the previous findings, our data showed that Dex inhibited the cell osteogenic potential, as evidenced by MTT, ARS staining, and EdU staining [33–35]. In addition, Western blot results demonstrated that Dex-incubated osteoblasts downregulated two key H_2_S-producing enzymes CBS and CSE. These findings support the potential role of H_2_S in osteoporosis. We further examined the effect of exogenous H_2_S on cell viability in osteoblasts treated with Dex. We found that GYY4137 (100 μM) treatment for 60 min dramatically alleviated Dex-induced cell damages, as indicated by the CCK-8 Cell viability analysis. Skeletal development and bone remodeling require stringent control of gene activation. The widely used indicators for osteogenic differentiation include Runx2, Osterix, and ALP. All these markers are related to bone formation [36, 37]. Here, we observed that GYY4137 significantly increased the osteoblastic marker levels in Dex-treated osteoblasts, suggesting that exogenous H_2_S alleviated Dex-inhibited osteoblastic differentiation. Furthermore, EdU assay by fluorescence microscopy further demonstrated that the GYY4137 attenuated the inhibitory effects of Dex on osteoblast proliferation.

Increasing evidence suggests that GCs could induce apoptosis of the osteoblasts and osteocytes [38–41]. The increased apoptosis of osteoblasts and the loss of osteocytes can result in disrupted osteocyte–canalicular network and failure to respond to bone damage [42]. Studies have reported that GCs induced apoptosis of the osteoblasts by activating caspase-3 and glycogen synthase kinase 3β [43, 44]. Our flow cytometry results demonstrated that treatment with Dex induced osteoblast apoptosis. These changes were significantly relieved when the cells were pretreated with GYY4137. This anti-apoptotic role of GYY4137 was further confirmed by the decreased expression of active Caspase-3 in the cultures. Cell apoptosis can also be modified by other apoptotic regulators including members of the BCL and Bax family [45]. In this study, we further observed the apoptotic factors BCL-2 and Bax expression. As we expected, the Western blot data showed that Dex caused a reduction in BCL2/BAX ratio in the cultures. However, this decrease was relieved in cells pretreated with GYY4137.

As an important modulator of bone formation metabolism, Wnt/β-catenin signaling pathway induces the generation of osteoblasts by stimulating the production and secretion of OPG [46–48]. Our previous work confirmed that GCs inhibited osteoblast proliferation by regulating Wnt/β-catenin pathway [49]. Wnt/β-catenin signaling also facilitated the transcription of CSE and promoted the formation of H_2_S [50], and H_2_S treatment activated the Wnt/β-catenin signaling [18, 51, 52]. In our work, pretreatment of Dex-incubated osteoblasts with exogenous H_2_S upregulated the Wnt/β-catenin signaling. The effect of H_2_S was attenuated after pretreatment with a Wnt/β-catenin inhibitor (XAV939). Furthermore, we found that knockdown of Wnt3a or Wnt6 abolished the GYY4137 effect on Dex-treated osteoblast proliferation and differentiation. Therefore, the Wnt/β-catenin pathway is likely involved in the protective effect of H_2_S against GIO activities.

## CONCLUSIONS

In this experimental study, we demonstrated that exogenous H_2_S attenuated Dex-induced inhibition of osteoblasts proliferation and osteogenic differentiation by activating the Wnt/β-catenin signaling pathway. Exogenous H_2_S also protected osteoblasts activities against Dex-induced apoptosis. These findings suggest that exogenous H_2_S is a promising option for the prevention and treatment of GCs-induced osteoporosis and osteonecrosis.

## MATERIALS AND METHODS

### *In vivo* treatment

All experiments involving animals were approved by the Medical Ethics Committee of the Second Military Medical University (SMMU). Three-month-old male Sprague-Dawley rats were obtained from the Laboratory Animal Center of SMMU (Shanghai, People’s Republic of China). All animals were housed in plastic pans (five animals per pan) and kept under standard laboratory conditions (12 hours of light, 12 hours of the dark; 25°C). The animals were allowed to acclimate to the animal facility for one week prior to the experiments.

During the first stage, the rats were injected intraperitoneally with dexamethasone (Dex) (Sigma, Louis, USA) mixed with sodium phosphate injection solution (5 mg/kg body weight) or saline once a day for 4 weeks. During the therapeutic stage, the rats were divided into four groups (five rats per group). The control group (saline only) was treated with either GYY4137 (Sigma, Louis USA) or vehicle. The Dex-induced group was treated with either GYY4137 or vehicle. The treatment was given as intraperitoneal injections of GYY4137/vehicle at 1 mg/rat every other day for 8 consecutive weeks. Mice in all groups were administered with tetracycline (30mg/kg; Sigma-Aldrich, St. Louis, MO, USA) 10 and 3 days prior to euthanasia to permit dynamic histomorphometry. Blood samples and bone tissues were collected after the rats were euthanized. Serum was separated by centrifugation and stored at –80°C until analyzed. Bone marrow stem cells (BMSCs) from the tibia and femur were flushed out with alpha-Modified Eagle’s Medium (α-MEM) (Hyclone, Logan, USA) and cultured in the growth medium [α-MEM containing 10% fetal bovine serum (FBS), 1% penicillin-streptomycin (Hyclone, Logan, USA)] at 37°C with 5 % CO_2_.

### Skeletal phenotyping

The distal end of the intact femurs was scanned using micro-computed tomographic (μCT) (GE Locus SP) to assess the bone mass, density, and trabecular microarchitecture. Parameters computed from these data included bone mineral density (BMD), trabecular number (Tb.N), Bone Volume/Total Volume (BV/TV), trabecular separation (Tb.Sp), and trabecular thickness (Tb.Th).

### Histological analysis

Femurs were fixed in 4% paraformaldehyde (Aladdin, Shanghai, China) and decalcified in EDTA-buffered saline solution (pH 7.4, 0.25 M) (Aladdin, Shanghai, China). Tissue sections were then cut longitudinally to obtain 5 μm sections. HE staining, TRAP staining, and Masson trichromic staining were followed to assess the histological changes. The histomorphometric examination included imaging using a Zeiss microscope (Carl Zeiss, Oberkochen, Germany).

### Dynamic bone formation

The tibias were obtained at sacrifice and fixed in 70% ethanol. The bone tissues were then dehydrated through a graded series of ethanol (70% to 100%), and embedded in methyl-methacrylate (MMA; Sigma-Aldrich, St. Louis, MO, USA) with 10% dibutyl phthalate (DBP) and 0.05% benzoyl peroxide (BPO). Tibias were cut, ground, and polished until a thickness of roughly 10 mm sections were achieved. The sections were then imaged using a fluorescent microscope (Carl Zeiss, Oberkochen, Germany). Histomorphometric measurements included single-labeled surface (sLS), double-labeled surface (dLS), and interlabel thickness (IrLTh). These data were used to calculate the mineral apposition rate (MAR = Ir.L.Th/7 days; mm/day).

### Immunohistochemistry

Femur sections were incubated with 3% hydrogen peroxide (Aladdin, Shanghai, China) for 10min and were blocked with 3% normal goat serum (Invitrogen, Paisley, UK) in Tris-buffered saline (Convance, New Jersey, USA). Next, the sections were incubated with rabbit anti-mouse Runx2 polyclonal antibodies (Santa Cruz Biotechnology, CA) at 4 °C overnight. Subsequently, biotinylated secondary antibodies were added to the sections, followed by a peroxidase-labeled streptavidin-biotin staining technique (DAB kit, Invitrogen, Paisley, UK).

### H_2_S, CTX-I and P1NP measurement in plasma

Plasma H_2_S, C-terminal telopeptide of collagen type 1 (CTX-1), and procollagen type 1 N-terminal propeptide (P1NP) levels were quantified using commercially available ELISA kits (mlbio, Shanghai, China) based on the manufacturer’s instructions. Briefly, a total of 100 μl samples were added to the microplate and incubated for 2 h. Then, equal volumes of the primary antibodies were added to each well and incubated for another 1 h, followed by the final incubation with horseradish peroxidase-conjugated secondary antibodies for 0.5 h. All the above incubations were performed at 37°C. The samples were washed 3 times with TBST after each incubation. The optical density (OD) values at 450 nm were determined using a fluorescence microplate reader.

### Cell culture

The primary osteoblast precursors were isolated from the calvaria bone of the newborn Sprague Dawley rats. These rats were obtained from the Laboratory Animal Center of SMMU (Shanghai, China). Cells were cultured in α-MEM supplemented with 10% FBS and 1% penicillin-streptomycin solution in a humidified culture chamber at 37°C with 95% air and 5% CO_2_. All the experiments were performed under differentiation conditions with 50 mg/mL ascorbic acid or 4 mmol/L β-glycerophosphate.

### MTT assay

The cells were seeded in a 96-well plate in the presence or absence of Dex (1 μM/0.1μM). After the treatment, 20 μl MTT was added to each well and incubated for 4 h at 37 °C. This procedure was followed by adding 150 μL dimethyl sulfoxide (DMSO) to dissolve the formed purple formazan dye. A scanning muti-well spectrophotometer (Multiskan MK3, Thermo Scientific, USA) was then used to measure the absorbance at 490 nm. The vehicle-treated control group was taken as 100% cell viability and all the other groups were normalized to this value.

### Cell counting kit (CCK-8) assay

The osteoblasts were inoculated at a density of 2 × 10^3^ per well into 96-well plates and cultured at 37°C with 5% CO_2_. CCK-8 measurement was performed according to the CCK-8 Kit instructions (Dojindo Laboratories, Japan). Briefly, 10 μl of WST-8 was added into each well at 37°C with 5% CO_2_ for 1 h. The absorbance of each sample was measured at a wavelength of 450 nm.

### Alkaline phosphatase and Alizarin red S staining

The cells were seeded onto 6-well plates at 1×10^5^ cells per well and cultured with differentiation medium for 7 days or 14 days. The cells were then subjected to alkaline phosphatase (ALP) staining or alizarin red S (ARS) staining. Briefly, the cells were washed three times with PBS, fixed with 4% paraformaldehyde for 15 min, and stained with ALP reagent (Beyotime, Shanghai, China) or 0.2 % ARS solution (Cyagen, Suzhou, China) for 30 min at 37 °C. The staining was repeated for at least 3 times independently.

### 5-ethynil-2′-deoxyuridine (EdU) incorporation assay

The cells were inoculated at a density of 2 × 10^5^ per well into 24-well plates and cultured at 37°C with 5% CO_2_. A total of 50 μM of EdU (Sigma–Aldrich, St Louis, USA) was then added into each well for 2 h. Next, the cells were fixed with 4% formaldehyde for 15 min, followed by permeabilization with 0.5% Triton X-100 for 20 min at room temperature. After washing the cells 3 times with PBS, 100 μl of 1X Apollo reaction cocktail was added to each well for 30min at room temperature. Subsequently, the cells were stained with Hoechst 33258. The EdU incorporation rate was expressed as the ratio of EdU-positive cells (red cells) to total Hoechst 33342-positive cells (blue cells).

### Alkaline phosphatase activity

The ALP levels were determined by Alkaline Phosphatase Assay Kit (Beyotime, Suzhou, China). Briefly, the cells were cultured with differentiation medium for 7 days. For ALP measurements, the cells were lysed using 100 μl RIPA lysis buffer, and the cell supernatant was collected into a 96-well plate. After co-incubation of the substrates and p-nitrophenol for 30 min at 37 °C, ALP activity was determined at the wavelength of 405 nm.

### Annexin V-Fluorescein isothiocyanate/propidium iodide double staining assay

Osteoblast apoptosis was measured by Annexin V–FITC/PI double staining assays. The annexin V-fluorescein isothiocyanate (FITC)/propidium iodide (PI) apoptosis detection kit was purchased from Sigma (St Louis, USA). Briefly, the cells were washed in 4°C PBS and resuspended in 500 μl binding buffer. This procedure was followed by staining with 10 μl annexin V and 5 μl PI in the dark for 15 min at room temperature. The cells were then analyzed by flow cytometry.

### Hoechst 33342 staining

Cells apoptosis was measured with Hoechst 33342 Staining (Beyotime, Suzhou, China). Briefly, the cells were fixed in 4% paraformaldehyde for 5 min and stained with 2.5 mg/ml Hoechst 33342 DNA dye. Uniformly stained nuclei were scored as healthy, viable cells. Condensed or fragmented nuclei were scored as apoptotic.

### Measurement of caspase-3 activity

Caspase activities were measured with a caspase activity kit according to the manufacturer’s instructions (BioVision, Mountain View, USA). The cells were washed with cold PBS, resuspended in lysis buffer, and left on ice for 15 min. The samples were then added to the reaction buffer containing Ac-DEVD-pNA, incubated for 2h at 37°C. The absorbance of yellow pNA, cleaved from its corresponding precursors, was measured using a spectrometer at 405nm. The fold increase in activity was calculated as the ratio between values obtained from the treated versus the untreated controls.

### Lentiviral transduction and oligonucleotide transfection

Osteoblasts precursors were inoculated into the 6-well tissue culture plates at a density of 1 × 10^5^ cells in α-MEM medium. Once the osteoblasts reached 50–70% confluence, the cells were infected with Wnt3a or Wnt6 shRNA lentiviral particles (Santa Cruz Biotechnology), respectively. The control cells were transduced with control shRNA Lentiviral Particles-A (Santa Cruz Biotechnology).

### Quantitative real-time PCR (qRT-PCR) analysis

Total RNA was extracted from the cells using TRIzol reagent (Takara Biotechnology, Japan). cDNA was synthesized using the PrimeScript™ RT Master Mix (Takara, Tokyo, Japan) by reverse transcription according to the manufacturer’s protocols. QRT-PCR was performed to amplify the cDNA using the SYBR Premix Ex Tag kit (Takara Biotechnology, Japan) and an ABI 7500 Sequencing Detection System (Applied Biosystems, Foster City, CA). The following cycling conditions were used: 40 cycles of denaturation at 95°C for 5 s and amplification at 60°C for 24 s. β-actin was used as the housekeeping gene and all the reactions were run in triplicate. The primers used were:5′-CCAGGACTTGGAGGTACAGC-3′ (forward) and 5′-TCGGCACTCTCTGGTAATGT-3′ (reverse) for CBS; 5′-ATAGTCGGCTTCGTTTCCTG-3′ (forward) and 5’-TCGGCAGCAGAGGTAACAAT-3′ (reverse) for CSE; 5′-GCACAAGGAGTGGACGAAGC-3′ (forward) and 5′-CCTTCCCTTTCTTGTTCTGTCC-3′ (reverse) for Ahr; 5′-AGAGTGAGCGGCAGAGCAAG-3′ (forward) and 5′-GTGGGGTAAGGGGAGGCATT-3′ (reverse) for Axin-2; 5′-TTTCTGGGACGACAAGGGTTC-3′ (forward) and 5′-CAGTGCGTCAATGTTCAAGTGC-3′ (reverse) for Nkd2;5′-CCGGATGAGCACATAGC CAA-3′ (forward) and 5′- TCTCTCCTCAACAGCCA CTCG-3′ (reverse) for TGFβ3; 5′- ACACCAATGGC GAGTCCTTC-3′ (forward) and 5′-CAGTTCTCATAC CGTTGCTCCAC-3′ (reverse) for Wsp; 5′- CAGGCT GTGCTATCCCTGTA-3′ (forward) and 5′- CATACCC CTCGTAGATGGGC -3′ (reverse) for β-Actin.

### Western blot analysis

Plasma proteins were extracted from the osteoblasts using 10% SDS–PAGE (Coring, China). The membrane was blocked with 5% BSA in Tris-buffered saline containing 0.1% Tween 20 (TBST) for 2 h at 37 °C and cultured with the primary antibody overnight at 4°C. The membrane was then exposed to the appropriate IRDye 800 CW-conjugated secondary antibodies for 0.5 h at 37 °C. Infrared fluorescence bands were visualized using an Odyssey infrared imaging system (LI-COR Bioscience, Lincoln, NE). The obtained bands were quantified with the Quantity One software (Bio-Rad, Hercules, CA). The ratios of the protein of interest and β-actin were calculated to determine the changes in protein levels.

### Statistical analysis

All statistical analyses were performed using SPSS version 19.0 (SPSS, Inc.). Data were collected from 3 or more independent experiments and were presented as mean ± standard deviation (SD). Data were analyzed using t-test or one-way ANOVA followed by Tukey’s test. Statistical significance was set at P < 0.05.
